# Human Consequences of Multiple Nuclear Detonations in New Delhi (India): Interdisciplinary Requirements in Triage Management

**DOI:** 10.3390/ijerph18041740

**Published:** 2021-02-11

**Authors:** Samir P. Desai, William C. Bell, Curtis Harris, Frederick M. Burkle, Cham E. Dallas

**Affiliations:** 1 Institute for Disaster Management, College of Public Health, University of Georgia, Athens, GA 30606, USA; dsamir01@outlook.com (S.P.D.); wcbell@uga.edu (W.C.B.); cuharris@uga.edu (C.H.); 2 Department of Health Policy and Management, College of Public Health, University of Georgia, Athens, GA 30602, USA; 3 Harvard Humanitarian Initiative, Harvard School of Public Health, Cambridge, MA 02138, USA; fburkle@hsph.harvard.edu; 4 Woodrow Wilson International Center for Scholars, Washington, DC 20004, USA; 5 Department of Emergency Medicine, Medical College of Georgia, Augusta University, Augusta, GA 30912, USA

**Keywords:** nuclear disasters, global health workforce, foreign medical teams, public health emergencies, world health organization, triage, India, interdisciplinary

## Abstract

The human casualties from simulated nuclear detonation scenarios in New Delhi, India are analyzed, with a focus on the distribution of casualties in urban environments and the theoretical application of a nuclear-specific triage system with significant innovation in interdisciplinary disaster management applicable generally to urban nuclear detonation medical response. Model estimates of nuclear war casualties employed ESRI’s ArcGIS 9.3, blast and prompt radiation were calculated using the Defense Nuclear Agency’s WE program, and fallout radiation was calculated using the Defense Threat Reduction Agency’s (DTRA’s) Hazard Prediction and Assessment Capability (HPAC) V404SP4, as well as custom GIS and database software applications. ESRI ArcGISTM programs were used to calculate affected populations from the Oak Ridge National Laboratory’s LandScan^TM^ 2007 Global Population Dataset for areas affected by thermal, blast and radiation data. Trauma, thermal burn, and radiation casualties were thus estimated on a geographic basis for New Delhi, India for single and multiple (six) 25 kt detonations and a single 1 mt (1000 kt) detonation. Major issues related to the emergency management of a nuclear incident are discussed with specific recommendations for improvement. The consequences for health management of thermal burn and radiation patients is the worst, as burn patients require enormous resources to treat, and there will be little to no familiarity with the treatment of radiation victims. Of particular importance is the interdisciplinary cooperation necessary for such a large-scale emergency response event, which would be exemplified by efforts such as the application of a Nuclear Global Health Workforce.

## 1. Introduction

Since their inception in 1947, the rivalry between India and Pakistan has resulted in four major military conflicts between the two nations in 1947, 1965, 1971, and 1999. The Kargil war of 1999 was particularly significant as it occurred under the shadow of nuclear weapons possessed by both India and Pakistan. Following the Cuban missile crisis, when the US and the Soviet Union came close to a nuclear weapon exchange, the importance of maintaining open lines of communication between nuclear powers was readily apparent, eventually leading to a substantial reduction in the nuclear arsenals of both nations. By contrast, repeated military conflicts between India and Pakistan have only led to an in-crease in rhetoric and brinksmanship. A meaningful cessation of nuclear rivalry between two states requires a vigorous process of confidence-building measures, negotiations, treaties and finally, a reduction in nuclear arsenals on both sides. Ominously, India and Pakistan have yet to embark on this journey of de-escalation.

The following sections present the methodology of analyses, derivation of the results, and a discussion of the impact of nuclear weapons’ detonations on New Delhi, India in terms of human casualties with an emphasis on injury distribution by blast pressure, thermal burns, and radiation exposure respectively. Mass casualty management of a nuclear detonation event is discussed along with the application of a nuclear event-specific triage system, with the need for a significant improvement in interdisciplinary collaboration between medical, public health, law enforcement, transportation, and national military assets.

This study aimed to evaluate the potential human casualty impact of a nuclear weapon detonation in New Delhi, India utilizing injury distribution by trauma (dictated by blast pressure), thermal burns, and radiation exposure. The application of a nuclear event specific triage system is then discussed using this data in mass casualty management decision-making. This approach is then discussed in terms of its demonstration of the distinct need for a significant improvement in interdisciplinary collaboration between medical, public health, law enforcement, transportation, and national military assets.

## 2. Materials and Methods

An overview of the effects of a nuclear detonation is provided below, followed by a description of the study area, weapon sizes, variable selection and modeling parameters. The DTRA HPAC v4.04SP4 [1] model approach is considered the most advanced nonclassified nuclear event casualty model system currently available, and was utilized to demonstrate injury category distributions for triage applications. From the various population estimate and GIS approaches available, the population estimates (in interpolated 3 arc-second grid format) were derived from Oak Ridge National Laboratory’s LandScan^TM^ 2014 Global Population Dataset [2], along with ESRI’s ArcGIS^TM^ software [3], as these are demonstrated as quite useful in HPAC.

### 2.1. Variable Selection and Model Used

The primary variables used to calculate human casualties in a nuclear detonation are blast, thermal, prompt and fallout radiation. Blast, thermal, and prompt radiation effects extend outwards in all directions from the point of detonation and are depicted as effects circles. Fallout radiation extends as a plume from the point of detonation and its course depends upon local atmospheric conditions such as wind direction. The approximate radii of impact for each effect circle for a 25 kt and 1 mt yield nuclear weapon respectively are shown in Table 1.

Blast effects for 0.6, 2, and 3 psi primarily consist of their impact on inert structures, whereas the impact of 4.9 and 8.1 psi overpressures were based upon the National Planning Scenarios [4]. 0.6 psi is the approximate threshold for glass breakage and is associated with secondary injuries such as cuts on exposed body surfaces due to wind blown glass and other debris. Therefore, the glass breakage zone will extend out the farthest in a concentric circle from Ground Zero (except for the narrow zone of the radiation plume). Two and three psi blast overpressures can cause tertiary injuries due to physical displacement of the body against inert structures and quaternary injuries owing to collapsed buildings. Individuals in the 4.9 psi effect circle are expected to suffer a 50% injury rate and those in the 8.1 psi effect circle, a 50% fatality rate.

Thermal effects consist of a 50% probability of 1st, 2nd, 3rd degree burns, and mass fires respectively. Table 2 shows the thermal fluence thresholds for burns based on Figure 12.65 of Glasstone and Dolan [5], and for mass fires based on Binninger’s work [6].

For the prompt radiation effect circle, a dose of 600 rads (6 Gy) with a 50% fatality rate was applied to the 25 kt and 1 mt weapons. Fallout radiation plumes were categorized according to the modified-SALT triage system for a nuclear detonation [7] and are shown in Table 3.

Fallout radiation isolines were calculated using DTRA’s HPAC v4.04SP4 [1]. The fallout radiation plumes after detonation were analyzed for affected populations after 4 and 24 h. Population estimates (in interpolated 3 arc-second grid format) were derived from Oak Ridge National Laboratory’s LandScan^TM^ 2014 Global Population Dataset [2]. ESRI’s ArcGIS^TM^ software [3] was used to create circular buffers around the detonation point representing regions where the population would be exposed to prompt radiation, blast and thermal effects. The population grid for Delhi was converted to polygon data (projected to UTM-WGS84 coordinates) and the population calculated for each intersection polygon of the blast, prompt radiation and thermal effects with the fallout radiation isolines. The polygon’s population was interpolated from the 3” Landscan population grid using the area of the intersection polygon divided by the relevant gridcell areas times that gridcell’s population. For each scenario the blast, thermal and prompt radiation circular zones of interest, HPAC plume fallout radiation isolines, and the population grid were overlaid. The affected population for each unique combination of blast, thermal and prompt radiation effects and fallout zone was tabulated using QlikTM software and assigned to the appropriate triage category using the modified-SALT triage system for a nuclear detonation event [7].

### 2.2. Effects of a Nuclear Detonation

The vast amount of energy from a nuclear detonation is released in the form of a blast wave (50%), thermal energy (35%), or nuclear radiation (15%). The blast wave is propagated outwards from the point of detonation and is measured as the amount of pressure over and above the normal atmospheric pressure (termed ‘overpressure’) in pounds per square inch (psi). The extent and intensity of the blast wave dissipates as it moves further away from the detonation site. The blast wave also generates very high velocity winds termed dynamic pressure, which can cause serious damage to structures. Taken together, overpressure and dynamic pressure can cause serious injuries due to the physical displacement of individuals into inert objects, collapse of solid structures such as buildings and trees, and glass projectiles from shattered windows. Damage to physical structures at different overpressures can manifest as follows [8]:

- 0.1 to 1 psi—Minor damage, mainly broken windows.
- 1 to 5 psi—Most buildings will sustain considerable damage in the form of blown out interiors and some may even collapse, unless they are constructed using reinforced concrete.
- 5 to 8 psi—Most buildings will be severely damaged or destroyed.
- >8 psi—Even heavily reinforced structures will be significantly damaged.

According to the 2011 population census, approximately 1.8 million of the 16.8 million inhabitants of Delhi are categorized as living in slums [9]. The slum areas consist of very flimsy physical structures constructed from tin sheets, cloth, wood, and in some cases, brick. Therefore, it is logical to assume that almost all slum dwellings will suffer severe blast damage, some even at 0.6 psi overpressure.

Thermal radiation (fluence) is typically measured in calories per square centimeter (cal/cm^2^). The impact of thermal fluence is a function of the weapon yield, the fraction of that yield released as thermal energy, distance from the blast site, and local atmospheric conditions [6]. Larger weapon yields result in an increased intensity and range of thermal effects [5]. The thermal impacts of a nuclear explosion are significant, particularly for weapons over 100 kt, scaling faster than blast, since thermal radiation decays as the inverse square while blast decays as the inverse cube of distance from the detonation point. Thermal energy travels directly from the fireball unless scattered or absorbed. A thermal fluence of 10 cals/cm^2^ or above is required to ignite buildings and other structures. Cloud cover is an important consideration in determining the effect of thermal energy. If the fireball from the detonation remains below cloud level, thermal radiation is reflected by the clouds and can amplify fire ignition probabilities [6]. Individual fires have the potential to coalesce and form mass fires. Based on the experiences of mass fires in Germany and Japan during World War II, the minimum requirements to produce mass fires include a burning area of half a square mile with half the structures on fire simultaneously, wind speed less than 8 miles per hour, and at least 8 pounds of combustible substances per square foot of the fire area [5].

Radiation consists of two main types, prompt and fallout. Prompt, or ionizing radiation, is the radiation emitted within one minute of a nuclear detonation. The prompt radiation zone lies within the mass fire zone for the 25 kt and 1 mt weapons evaluated in this study. In a surface burst, large quantities of soil are sucked into the fireball along with the weapon casing and other materials present in the vicinity of the explosion. The high temperatures within the fireball can either fuse or vaporize these substances which are subsequently contaminated with radioactive fission fragments produced during the nuclear detonation. The contaminated particles gradually descend to the ground, a phenomenon called radioactive fallout. Radioactive fallout is the main source of residual nuclear radiation and manifests itself in the form of gamma rays and beta particles. Some of the heavier fallout particles descend quickly to the earth (within the first 24 h after a nuclear explosion) and are called local and/or early fallout. Fallout radiation causes a conical shaped plume that is blown downwind from the blast site. Dispersion is greatly affected by turbulence in the atmosphere which in turn mainly depends upon the topography, land use, vertical wind and temperature structure. Fallout radiation, measured in Gray (Gy), has been partitioned into five classes (≤0.5 Gy, >0.5–2 Gy, >2–6 Gy, >6–10 Gy, >10 Gy) based upon the modified-SALT triage categories of radiation injury [7].

### 2.3. Study Area and Size of Weapons

**Figure 1:**
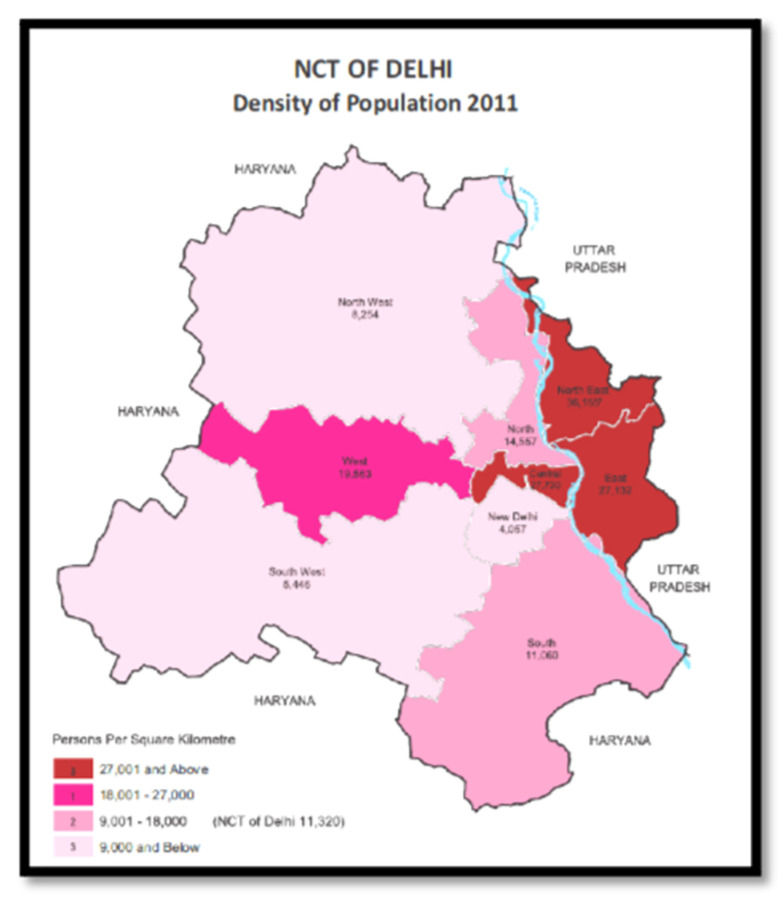
Population Density—National Capital Territory (NCT) of Delhi.

New Delhi is one of the 11 districts that comprise the National Capital Territory (NCT) of Delhi (Figure 1). According to the latest census data in 2011 [9], the population of the NCT of Delhi is approximately 16.8 million, increasing at 2.09% annually [10]. Population density is high with 11,320 individuals per square kilometer as shown in Figure 1 [11]. The physical topography of Delhi is relatively flat. The month of May was selected as the time of the nuclear attacks as it is one of the driest months with little cloud cover and is therefore most amenable to dispersion of fallout radiation and thermal energy.

Three separate scenarios were selected for the use of nuclear weapons. First, a single 25 kt weapon was detonated in the center of New Delhi where some of the most important Indian government institutions (such as the Indian parliament) are located. This could either be a highly efficient fission weapon or a less efficient boosted weapon. A height of 25 m was selected to ensure that fallout radiation was high, while thermal effects are still substantially greater than a surface burst. In the second scenario, six 25 kiloton (kt) nuclear weapons were detonated at the same height at six separate locations throughout Delhi in such a manner as to cover the major population clusters in the city, as would be expected to ensue with modern nuclear war planning conducted by all states possessing nuclear weapons., and in the third scenario, a one megaton (mt) nuclear weapon at a height of 200 m was detonated. It is recognized that neither India nor Pakistan has thermonuclear weapons at this time, but this will likely become true within the next decade. A fission fraction of 1 was assigned to the 25 kt weapons and 0.8 for the 1 mt device.

**Figure 2:**
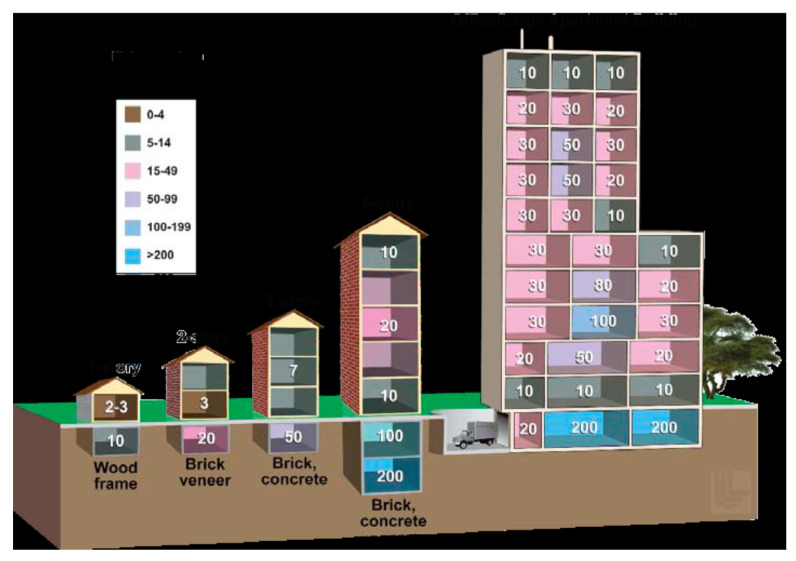
Protection Factors by building type and location within the building.

The six 25 kt nuclear detonations were simulated at various locations There are some common aspects in each of the three scenarios which should be considered while reviewing the results. Since May is one of the hottest months in Delhi, most people will be dressed in light summer clothes and it can be safely assumed that any protection offered by clothing to even minor burns will be minimal. Also, buildings and other concrete structures may offer varying degrees of shielding, particularly from fallout radiation. The level of shielding is denoted by a protection factor (PF). For example, a structure with a PF of 10 indicates that a person inside the building will receive 1/10th of the radiation dose compared to a person out in the open. The PF also depends on the location of a person within the building. A summary of PFs by building type and location within the building is provided in Figure 2.

PFs have not been considered in the calculation of human casualties due to the significant diversity in the type of structures (slums, single-story houses, skyscrapers) that exist within the affected areas. Therefore, in a real-world setting, it is conceivable that some individuals may be exposed to less or no fallout radiation at all, while others in slums or in outdoor settings will have relatively higher exposure to radiation without appreciable shielding.

**Figure 3:**
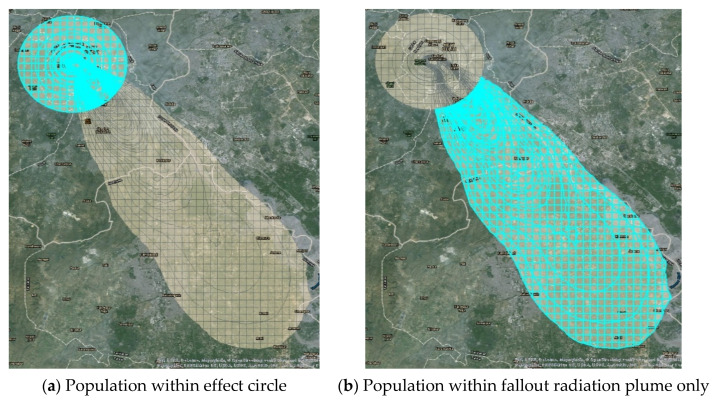
Calculation of human casualty data.

The highlighted areas in Figure 3a,b show the methodology behind calculation of human casualty data. Figure 3a contains the population affected by blast, thermal, prompt, and fallout radiation, and Figure 3b shows the population affected by fallout radiation only. The number of casualties within the effect circles (Figure 3a) are calculated based on the various combinations of injuries that people are likely to experience along with the associated fallout radiation, if any.

**Figure 4:**
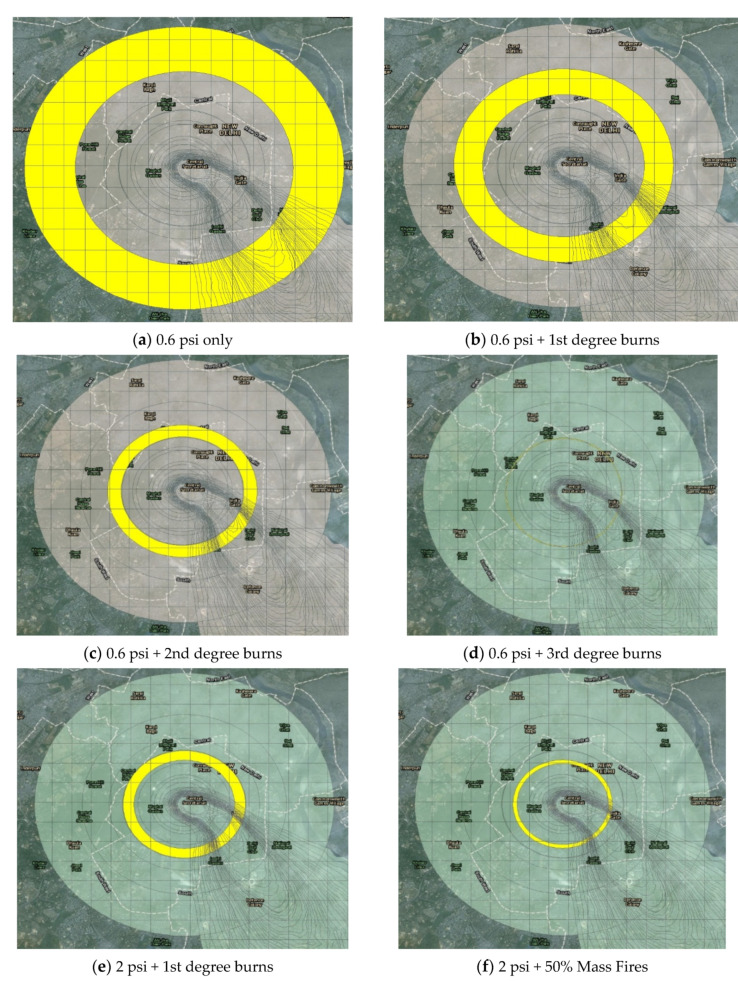

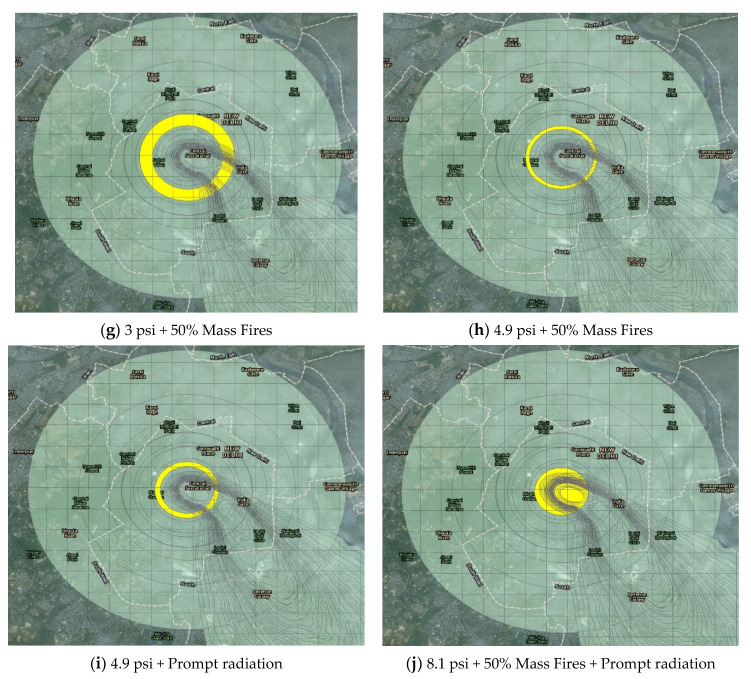
Effect circles and their associated impacts for a single 25 kt nuclear weapon.

Figure 4 shows the types of impacts from the outermost 0.6 psi ring to the innermost 8.1 psi effect circle. The effect circles containing thermal injuries (1st, 2nd, 3rd degree burns and mass fires) indicate a 50% probability of such injuries occurring in the population. Populations extending from the 2 psi + 50% mass fires ring (Figure 4f) to the innermost 8.1 psi + 50% mass fires + prompt radiation (Figure 4j) ring are considered beyond the reasonable ability of emergency response personnel to reach these patients, and therefore they are not capable of being triaged. The significant amount of structural damage (collapsed buildings, broken power lines and water pipes) and the possibility of mass fires in these areas will greatly reduce the probability of emergency workers being able to reach the survivors in time. Therefore, only the impacts depicted in Figure 4a–e will be categorized according to the modified-SALT triage system. Individuals exposed to only fallout radiation (Figure 3b) are grouped based on their level of radiation exposure into the appropriate modified-SALT triage categories for radiation injuries.

## 3. Results

Based on the assumption that resource availability for medical treatment of victims will be poor, subsets of the affected population are assigned to triage categories of minimal, immediate, or expectant. It is important to note that due to the expected very poor resource availability, the triage category of ‘delayed’ is not utilized as there is virtually no likelihood of distribution of resources beyond the immediate categories under current expectations of nuclear war preparedness.

The fallout radiation plume moves and spreads over time according to the prevalent wind direction. The populations affected by the fallout plume at 4 and at 24 h post-detonation respectively are enumerated in the subsequent sections. However, it must be understood that population groups at 4 and 24 h are neither mutually exclusive nor are they entirely congruent with each other. For example, a person experiencing fallout radiation of 0.5–2 Gy at 4 h could very well be subjected to fallout radiation levels of 2–6 Gy at the 24-h mark. However, the fallout plume also spreads by the 24-h period and will cover additional people as compared to the 4-h plume. Therefore, the 24-h group will contain some individuals that were also included in the 4-h group.

### 3.1. Single 25 kt Nuclear Weapon

Medical casualties from a 25 kt nuclear weapon detonation in New Delhi are presented in Table 4 and Table 5.

Of the approximately 3.36 million individuals that lie within the effect circles, 2.9 million (87.4%) will be eligible for triage. 423,175 (12.6%) people confined in the 2 psi + 50% Mass Fire (MF) zone up to the detonation point are considered not accessible for triage. 2.12 million (63%) could experience some combination of 0.6 psi blast overpressure, fallout radiation exposure up to 2 Gy, and a 50% probability of 1st degree burns. Injuries associated with these types of trauma include mild prodromal symptoms (nausea, vomiting, and lethargy) due to radiation exposure and, cuts and lacerations due to glass breakage. Therefore, these individuals will be assigned to the triage category ‘minimal’ (green) with access to medical care only as available after the immediate medical category. It should be kept in mind that these designations are inevitably used due to the very limited medical resources, not due to the actual needs of these patients.

An estimated 576,000 (17%) individuals that lie within the effect circles may be subjected to blast overpressure ranging from 0.6 to 2 psi, fallout radiation exposure up to 6 Gy, and a 50% probability of 2nd or 3rd degree burns. Although this population has been assigned to the ‘immediate’ (red) category, multiple factors will determine their actual triage outcome. The amount of total body surface area (TBSA) covered by the 2nd and 3rd degree burns will dictate whether the victim is triaged as immediate or ‘expectant’ (black). Additionally, the amount of fallout radiation exposure will also be a significant factor in determining the triage category. Individuals suffering from 2nd or 3rd degree burns covering <20% TBSA and fallout exposure up to 3 Gy may have a fair chance of survival if they receive medical care and will be assigned to the immediate triage category. However, people with 2nd or 3rd degree burns covering >20% TBSA and/or fallout radiation exposure greater than 3 Gy will likely be assigned to the expectant category. Furthermore, survivors in the 2 psi overpressure area would have to self-transport themselves to the triage facility as destruction of physical infrastructure would make it almost impossible for emergency workers to reach these individuals. Finally, about 233,000 (7%) of the individuals will suffer from fallout radiation exposure greater than 6 Gy, blast overpressures ranging from 0.6 to 2 psi, and a 50% probability of 1st, 2nd, or 3rd degree burns. Under poor resource conditions, victims with such complicated combined injuries and radiation exposure would be assigned to the expectant triage category.

**Figure 5:**
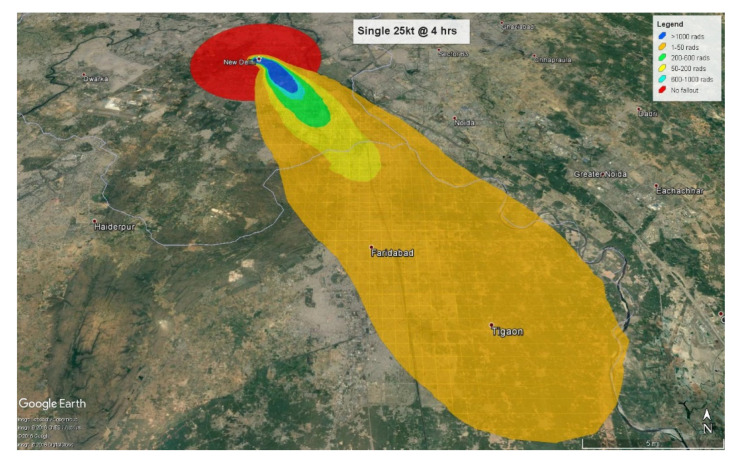
Combined injuries by triage categories.

The number of individuals affected only by fallout radiation are calculated at 4 and 24 h after detonation respectively. At 4 h, the fallout radiation plume will extend almost 28 km (17 miles) into the neighboring state of Haryana with some amount of fallout radiation spilling over into another neighboring state, Uttar Pradesh (Figure 5).

**Figure 6:**
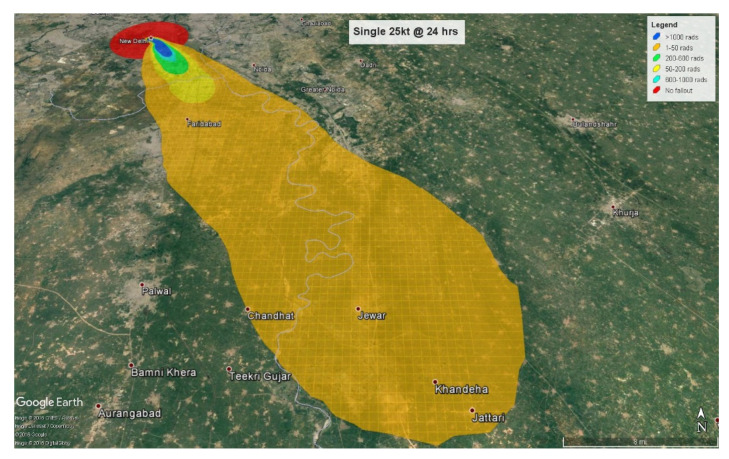
Fallout exposure (4 h) by triage categories.

At 24 h, the plume crosses Haryana and extends almost 21 km (13.5 miles) into Uttar Pradesh (Figure 6). Therefore, at the 4-h mark, 2,278,584 (80.6%) people will be triaged as minimal with 1,758,000 exposed to less than 0.5 Gy and 520,000 to 0.5–2 Gy. Everyone triaged to the minimal category will be expected to receive little or no treatment. Approximately 484,000 (17.1%) will be triaged as immediate, and 64,000 (2.3%) as expectant based on their radiation exposure levels of 2–6 Gy and greater than 6 Gy respectively. The immediate category will be expected to express a moderate level of radiation injury whereas the expectant group will have received fatal radiation doses. At 24 h after detonation, the fallout plume affects 3.8 million people with approximately 3 million (80.5%) exposed to radiation levels ranging from 0.01–2 Gy, categorized as minimal. The 565,000 (14.8%) individuals calculated as receiving 2–6 Gy will be categorized as immediate, and 177,000 (4.7%) receiving more than 6 Gy will be assigned to the expectant category.

### 3.2. Six 25 kt Nuclear Weapons

Medical casualties from six 25 kt nuclear weapon detonations over various locations in Delhi are shown in Table 6 and Table 7. The six detonation points were strategically selected to cover the major population centers in Delhi, as would be expected to occur with most nuclear war planning. Of the 8.46 million individuals that lie within the effect circles, 6.95 million (82%) will be eligible for triage and 1.5 million (18%) people are considered not accessible for triage. Approximately, 2.36 million (27.8%) individuals will be assigned to the minimal category, 2.7 million (32.8%) to immediate, and 1.8 million (21.5%) to the expectant triage category.

**Figure 7:**
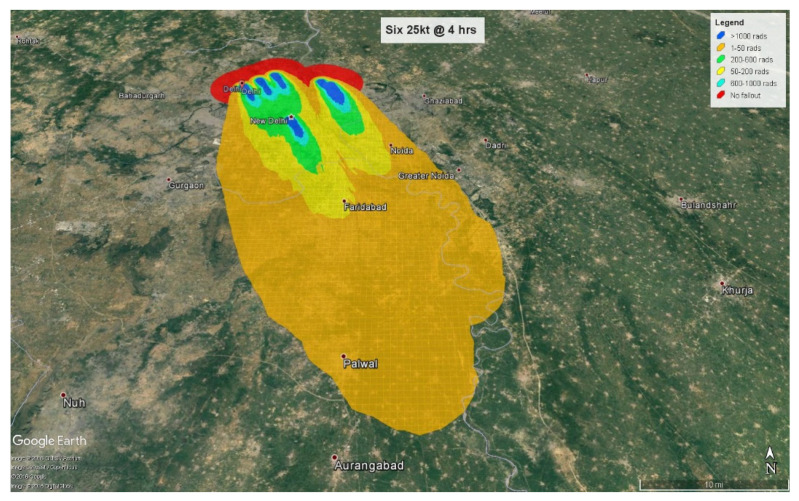
Fallout exposure (24 h) by triage categories.

**Figure 8:**
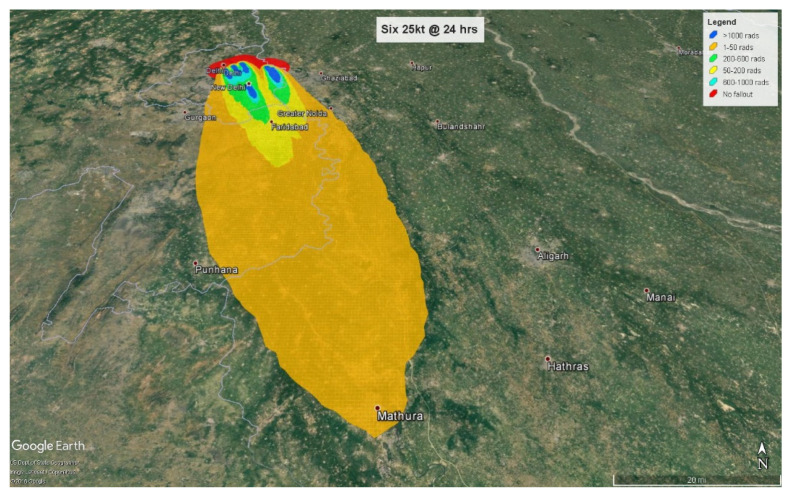
Single 25 kt fallout radiation plume at 4 h.

At 4 h, the fallout radiation plume will extend almost 50 km (31 miles) into the neighboring state of Haryana with some amount of fallout radiation spilling over into another neighboring state, Uttar Pradesh (Figure 7). At 24 h, the plume crosses Haryana and extends almost 54 km (33 miles) into Uttar Pradesh (Figure 8).

Of the 10.4 million people affected by fallout radiation at 4 h post-detonation, 6.84 million (65.6%) people will be triaged as minimal, with 3.5 million exposed to less than 0.5 Gy and 3.34 million to 0.5–2 Gy. 2,768,000 (26.5%) will be triaged as immediate, and 816,000 (7.9%) as expectant based on their radiation exposure levels of 2–6 Gy and greater than 6 Gy respectively. At 24 h, the fallout plume affects approximately 13.2 million people from which approximately 8 million (60.6%) individuals will be exposed to radiation levels ranging from 0.01–2 Gy and categorized as minimal. 2.76 million (23%) individuals receiving 2–6 Gy will be categorized as immediate, and 2.16 million (16.4%) receiving more than 6 Gy will be assigned to the expectant category.

### 3.3. Single 1 mt Nuclear Weapon

The amount of human devastation caused by a 1 mt nuclear weapon on New Delhi is extraordinary and is shown in Table 8 and Table 9. The effect circles for a 1 mt nuclear detonation not only cover most of Delhi, but also encompass areas in the neighboring states of Haryana and Uttar Pradesh. Of the 18.7 million people that may be expected to have injuries within 4 h, slightly more than 13 million (70%) are eligible for triage whereas the remaining 5.5 million (30%) would not be accessible for triage. From the triageable population, 6.68 million (36%) are assigned to minimal, 4.94 million (26%) to immediate, and 1.55 million (8%) to expectant.

**Figure 9:**
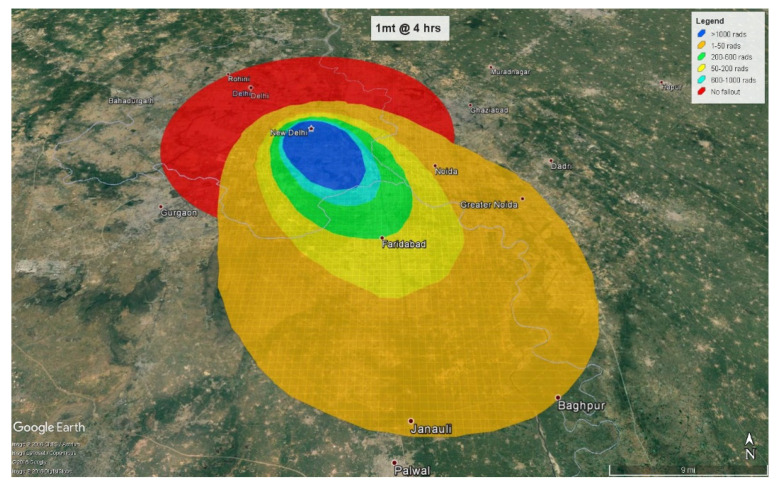
1 mt fallout radiation plume at 4 h.

**Figure 10:**
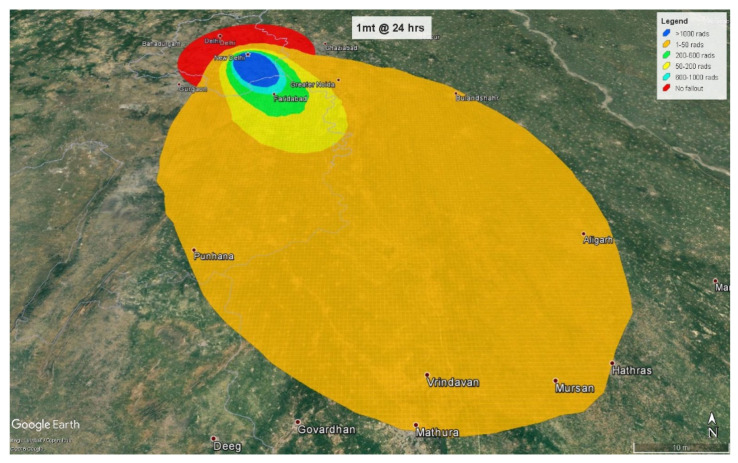
1 mt fallout radiation plume at 24 h.

At 4 h, the fallout radiation plume will extend almost 38 km (24 miles) into the neighboring state of Haryana with some amount of fallout radiation spilling over into another neighboring state, Uttar Pradesh (Figure 9). At 24 h, the plume crosses Haryana, extends almost 78 km (48 miles) into Uttar Pradesh, and spreads to Haryana’s neighboring state of Rajasthan (Figure 10).

Of the 2.2 million people affected by fallout radiation at 4 h post-detonation, 2.12 million (96%) people will be triaged as minimal. 87,780 (4%) will be triaged as immediate based on their radiation exposure levels of 2–6 Gy. Interestingly, in this thermonuclear detonation, the fallout radiation levels greater than 6 Gy lies completely within the effect circles and therefore, there are no individuals affected only by fallout radiation greater than 6 Gy. Consequently, there are no expectant individuals at 4 h, that is, there are no surviving patients who are receiving an eventually lethal dose of radiation without other life-threatening injuries. At 24 h, the fallout plume affects approximately 10.5 million people from which approximately 9.4 million (89.5%) individuals will be exposed to radiation levels ranging from 0.01–2 Gy, categorized in the minimal triage category. Slightly more than one million (10%) individuals receiving 2–6 Gy will be categorized as immediate, and 66,000 (0.5%) individuals receiving more than 6 Gy will be assigned to the expectant category.

### 3.4. Comparison of 25 kt, 6 × 25 kt, and 1 mt Casualties

**Figure 11:**
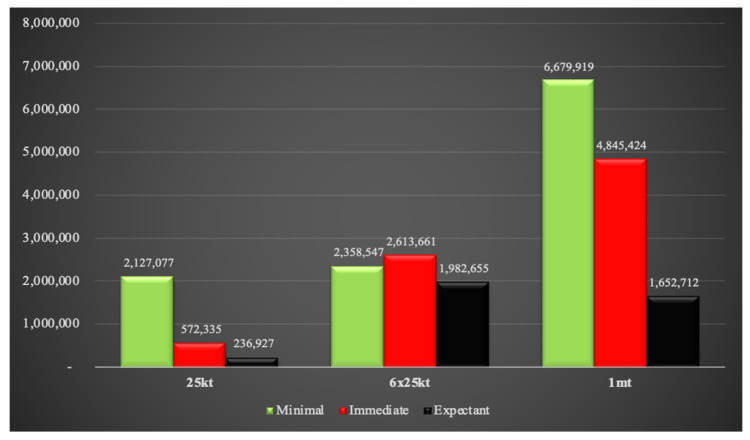
Combined injuries based on triage assignments.

The number of combined injuries based on triage assignments for all three weapon yields are shown in Figure 11. Interestingly, the number of individuals requiring minimal medical care are similar between the single 25 kt and the 6 × 25 kt weapons. However, there is an exponential rise in the immediate and expectant triage category numbers between these two weapon sizes. The difference between 6 × 25 kt and the 1 mt weapons is even more dramatic for the minimal and immediate triage categories. However, the expectant population in the 1 mt scenario is less than that in the 6 × 25 kt setting. This is likely due to the intentional inclusion of major population clusters for the 6 × 25 kt simulation compared to the 1 mt detonation which is simply geographically located in the center of New Delhi. Therefore, the number of expectants may increase significantly if large population groups are specifically targeted by the 1 mt weapon by nuclear war planners rather than political targets. The populations requiring immediate medical attention in the 6 × 25 kt and the 1 mt situations will be massive, approximately 2.7 million and 4.9 million respectively. It is virtually certain that sufficient human and medical resources would not be available to care for such large numbers of people.

**Figure 12:**
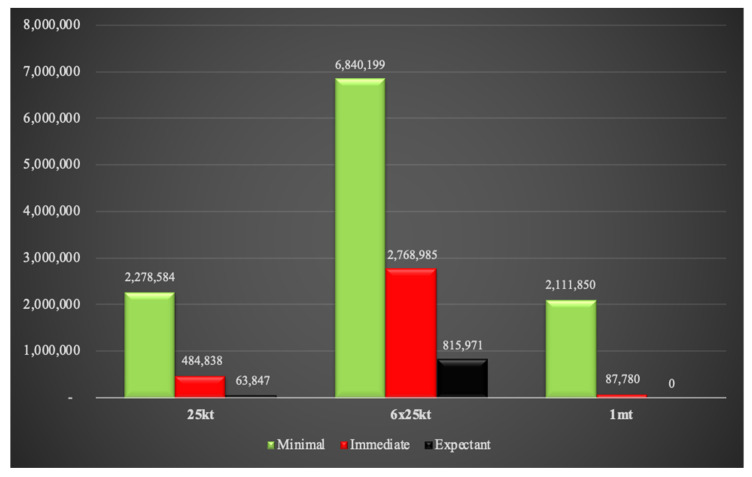
Fallout radiation exposure at 4 h.

**Figure 13:**
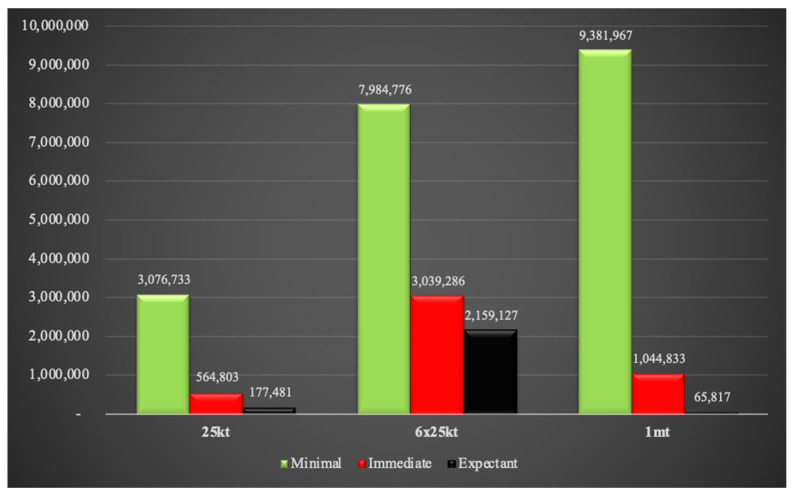
Fallout radiation exposure at 24 h.

Fallout radiation exposures at 4 h and 24 h based on triage assignments are provided in Figure 12 and Figure 13. Individuals exposed to 2–6 Gy of fallout radiation are categorized as requiring immediate medical attention. At 4 h, almost half a million individuals will be categorized as immediate in the single 25 kt simulation compared to 88,000 people in the 1 mt situation. This anomaly is because at 4 h, most of the 2–6 Gy radiation plume in the 1 mt weapon falls within the blast and thermal zones. At this time point, only a few people are exposed solely to fallout radiation for the 1 mt weapon compared to the single 25 kt weapon. The above-mentioned situation is reversed 24 h after detonation as the 1 mt fallout plume spreads over a greater area than the 25 kt plume. The 6 × 25 kt weapons scenario contains the maximum number of people affected by 2–6 Gy of radiation, but in contrast to combined injuries, many of these people could potentially be spared by swift evacuation away from the radiation plume.

## 4. Discussion

Evaluation of injury category distributions following an urban nuclear detonation has considerable utility in mass casualty management, as illustrated in this examination of a relatively small nuclear device in New Delhi, India. Of course, societal impacts will be catastrophic, and this will impact on emergency response capacity. In the current scenario, each of the three simulation models for a nuclear attack on New Delhi will have a direct impact on the central government infrastructure of India with subsequent governmental and healthcare response paralysis.

Perhaps one of the most compelling factors in this outcome is the effect of construction quality on casualty production. For example, single 25 kt nuclear simulation will involve central, south, and south-west Delhi which are some of the more affluent areas of the city. It is probable that buildings and houses in these areas are constructed using modern methods and materials and may offer some protection from the 0.6 psi blast overpressure, although there may be significant injuries from glass breakage. The six 25 kt and 1 mt scenarios will also involve some of the most densely populated areas in the city, including slum areas. The main concern from a 0.6 psi blast wave in slums is the potential for decapitating traumatic injuries. Slum construction consists of materials such as sheet metal for roofs which can easily turn into deadly projectiles from a 0.6 psi blast wave. Consequently, although the number of victims in the minimal triage category are almost similar in the single 25 kt (2.1 million) and the six 25 kt (2.3 million) scenarios, it is possible that individuals categorized as minimal may present with more serious traumatic injuries than expected in the latter simulation. The 1 mt weapon will cover more than two-thirds of Delhi, increasing the number of individuals in the minimal triage category (6.6 million) almost three times compared to the 25 kt simulations.

Urban nuclear detonations can be expected to result in devastating impacts on many societal functions, including the focus of this article with emergency response capability, and yet including many parallel contributing decrements such as transportation, food availability, security issues, electricity grid collapse, and availability of potable water. Each of these societal function losses will contribute to the difficulty in the immediate mass casualty emergency response to the detonation, and in the ensuing days and weeks afterward will contribute many other medical and public health needs which would have to be addressed. It is a sobering fact that no city in the world is prepared for this outcome, whether it is New Delhi, Karachi, New York, London, Moscow, or Beijing, and this reality calls for a planned international response to wherever this event first occurs.

In the current scenario, there is a massive increase in the number of victims assigned to the immediate triage category, from approximately 0.5 million (single 25 kt weapon) to 2.7 million (six 25 kt weapons) to 4.9 million (1 mt weapon). Structures in the more affluent areas impacted by the single 25 kt weapon may offer some protection against 2nd and 3rd degree burns, reducing the number of people requiring immediate medical attention. The reverse may be true in the six 25 kt and 1 mt situations as the flimsy slum construction will not provide the same level of protection from burn injuries. The highest number of victims in the expectant triage category due to combined injuries are observed in the six 25 kt simulation (1.8 million). However, the 1 mt simulation contains the highest number of individuals considered inaccessible for triage (5.5 million) which would include a significant number of fatalities.

Of the three separate simulations, the 1 mt weapon will result in the maximum number of combined injuries, whereas the six 25 kt weapons will affect the highest number of people due to the fallout radiation plume, similar to findings in other studies of multiple nuclear detonations [12]. Despite the greater spread of the fallout plume in the 1 mt weapon at 24 h, only about 1 million individuals could be exposed to 2–6 Gy requiring immediate medical attention compared to approximately 3 million people in the six 25 kt scenario.

Emergency management of nuclear detonations in an urban setting is influenced by a number of factors such as availability of medical personnel and resources, evacuation capabilities, and dissemination of information to the general public regarding the incident [13]. Even in the minimal triage group, it can be expected that there will be millions of people that will require basic care such as first aid and debridement of wounds. One of the solutions that have been proposed to the obvious workforce need for such casualty numbers is to conserve the limited number of medical personnel for those requiring intensive care, by arranging for additional training of ancillary healthcare groups such as dentists and veterinarians in mass casualty areas such as burn triage and wound debridement [14]. Another large-scale possibility for viable training improvement is to mobilize the existing large number of medical, dental, nursing, pharmacy, and veterinary schools around the country to train students in burn triage and mass casualty approaches to first aid procedures. As in many other areas of emergency management, a high degree of planning will be needed particularly in the area of the deployment of medical personnel and emergency workers to the affected areas. An interesting example of this has been the Radiation Triage, Transport, and Treatment (RTR) system developed by a US federal interagency working group to guide emergency operations after a nuclear detonation event. As this is one of the most demanding scenarios for mass casualty management, the RTR model provides efficient organization and deployment of personnel and resources across a broad range of casualty demands [15]. One unique example of the issues addressed is the past practice of locating RTR1 sites as close as possible to the blast epicenter, which has had the highly hazardous and unwanted result of deploying personnel within dangerous radiation fields. To help protect the health of an already challenged workforce this has led to the utilization of the latest protective action guidelines (PAGs) published by the US Environmental Protection Agency for emergency workers in a radiological environment, where it is mandated that these response personnel must not have radiation exposure levels over 0.25 Gy for life-saving interventions [16]. Therefore, in the current casualty prediction scenarios this would lead to the establishment of RTR1 sites at the inner periphery of the 0.6 psi and 1st degree burn zone adjacent to (but not in) the fallout plume. Specialized teams equipped with radiation monitors, personal protective equipment such as respiratory protection (N95 respirator or higher), and universal precautions such as gloves, light weight covering (to keep off radioactive dust), and booties [17] would decontaminate patients coming out of the radioactive areas and treat patients. It is unfeasible to actually send personnel into contaminated areas to gather more patients in any event, as the RTR1 sites are expected to have overwhelming numbers of immediate category patients even when sited in the inner periphery of the 0.6 psi zone. Only when the casualty care needs in the radiation periphery are met is it feasible to consider even brief entry into the fallout plume to rescue additional survivors. RTR2 sites will cater to the victims requiring minimal medical care, congruent with the minimal category of triage care. It is proposed to locate RTR2 sites on the outer perimeter of the 0.6 psi plus 1st degree burn zone adjacent to (but not in) the fallout plume. In conjunction with this maximum treatment with maximum safety approach, RTR3 sites would be located away from the prompt and fallout radiation zones. Most victims at RTR3 sites will be ambulatory with minor injuries and insignificant radiation exposure. It is a critical feature of these categorizations that nearly all of the public and a large portion of the emergency response community is likely to consider any detectable radioactivity as significant. However, the scientific consensus is that from a healthcare point of view, low levels of exposure are not expected to result in long term effects, and incident command must adhere to this reality in order to continue to be maximally efficient in the coordination and deployment of resources under these demanding conditions. Major activities at these sites will involve symptomatic treatment and evacuation of victims. Therefore, RTR3 sites can be located just outside the blast and fallout zones to assist in evacuation efforts.

Triage and treatment of wounded survivors should be complimented by evacuation efforts, particularly for populations projected to be in the path of the fallout radiation plume [13]. Three major factors will influence evacuation of victims. The projected path of the fallout plume along with its extent of coverage should be identified as soon as possible. Simultaneously, potential evacuation routes should be quickly disseminated to emergency workers. Finally, many people in the fallout plume will self-evacuate and will have to be decontaminated, and then evaluated for the appropriate triage category once they reach an RTR site. Sheltering-in-place is a doctrinal approach used in many urban areas, but the selected shelter needs to provide adequate radiation protection as well as offer adequate water and security. An adequate shelter is defined as one which ‘protects against acute radiation effects, and significantly reduces radiation dose to occupants during an extended period’ [8]. Concrete, brick, and stone act as good shielding materials and therefore, basements, underground garages, and tunnels are considered adequate shelters capable of reducing fallout exposure dose by a factor of 10 or more [8]. However, having a water supply is likely to be problematic without foresight to that issue, and security for vulnerable populations is highly likely to be a serious problem.

Forward planning is required in order to create stockpiles of medical supplies that can be rapidly mobilized in case of a nuclear event, though this has not actually occurred to a significant extent in most urban areas. Dual-utility therapeutic materials with extended shelf-lives include medical supplies that can be used in mass casualty situations as well as for routine healthcare applications. Cytokines, antibiotics, anti-emetics, and many other products are considered dual-utility [18]. National medical stockpiles should be strategically located away from the most likely target areas (i.e., downtown buildings and military installations), and have adequate security (in particular for narcotics) to afford some degree of survivability in the chaotic initial hours following a nuclear attack.

Healthcare facilities that remain functional will have to be prepared to receive large numbers of wounded people requiring additional treatment. Most of the major government hospitals are located in Delhi proper and it is assumed that almost all of these institutions will become non-operational after a nuclear attack. Therefore, the burden of treating nuclear mass casualty victims will fall on some of the large private healthcare institutions that are peppered all over the outskirts of Delhi within the National Capital Region (NCR). The government could reinforce mass casualty preparedness in these institutions by offering training and education to their staff on nuclear triage and wound management because reassessment of initial triage assignments would be needed to identify patients with the greatest chances of survival. The psychological impact of a nuclear attack on medical personnel is an important consideration. Research has also shown that emergency responders are less familiar with situations involving nuclear/radiological exposure compared to other types of emergencies, highlighting the need for nuclear-specific training and education [19].

India’s National Disaster Management Authority (NDMA) which is analogous to the US Federal Emergency Management Agency, has published guidelines for the management of nuclear and radiological emergencies including nuclear attacks [20]. Although specific actions and standard operating procedures to be undertaken in the event of a nuclear attack remain classified, the NDMA guidelines do provide some insight into nuclear incident preparedness at the national level. A network of 18 Emergency Response Centers (ERCs), each equipped with protective gear and radiation monitoring equipment, have been set up across India to respond to any type of nuclear emergency. The ERCs are also tasked with providing timely guidance and advice to first responders. Additionally, basic training on nuclear-related events is being imparted to the National Disaster Relief Force (NDRF) teams and to Quick Reaction Teams (QRTs) of the Indian paramilitary forces. The Bhabha Atomic Research Center (BARC), India’s premier nuclear research facility, has developed a host of ‘smart’ radiation monitoring systems with impact assessment capabilities which can quickly scan and monitor a contaminated area and present the outcomes in the form of a color-coded map. The Indian Defense Research and Development Organization (DRDO) has invested significant resources in developing detection equipment such as high-range radiation monitors for field use, personal dose monitors, and mobile systems such as nuclear field laboratories.

Interestingly, the NDMA guidelines also include a section on ‘gap analysis’ that elaborates on issues that remain to be adequately addressed in the nuclear/radiological domain. One of the primary issues identified in the gap analysis is the need for education, awareness, and training. The report concedes that currently the national education system does not include any instruction on nuclear/radiological emergencies and that the general public has very limited awareness of such issues. The report goes further claiming that “Even the intelligentsia have misconceptions about nuclear energy in general” [20]. Disaster management agencies will almost certainly face a lack of sufficient manpower and resources in case of a nuclear detonation. Therefore, creating awareness among the general public is probably the most efficient and cost-effective method in terms of preparedness for nuclear emergencies. However, communicating time-sensitive evacuation and safety information to the general public in the event of a nuclear disaster is critical and can only be accomplished via electronic media. It has been noted that while television has spread only to about 61% of Indian households, market penetration of public and commercial radio broadcasters is almost nationwide. The radio is still the primary means of disseminating electronic information in many rural parts of India and should be duly utilized to circulate official instructions in case of a nuclear emergency.

Another issue that creates additional obstacles to an appropriate response is the paucity of basic infrastructure such as good roads and effective disaster management communication systems. Currently, Indian roadways are ill-equipped to provide egress for a large number of people thereby hindering any potential evacuation efforts. Disaster management communication links between the local, state, and federal levels are thought to be neither dedicated nor adequate. However, the NDMA has taken concrete action in recent years to redress the communications issue by creating a dedicated National Disaster Communications Network (NDCN) which will provide fail-safe communications capability during disasters [21]. The need to identify potential locations where people can take shelter during a nuclear emergency is also mentioned in the gap analysis, though as in the United States, it is dubious that action has been taken in major urban areas. Other areas for improvement include creating a pool of radiological safety officers at the national level, strengthening the medical response mechanism, and creating disaster management plans specifically for some of the major Indian cities.

The proposed nuclear global workforce framework must include medical support to triage, care to those with the opportunity to survive, palliative care to the expectant population as well as the less-affected populations and those evacuated to safer ground [22]. Nuclear Triage Centers (NTCs) would be for centrally coordinated mobile and fixed initial triage and dose monitoring facilities designed to identify, assess, transfer, decontaminate and move casualties efficiently to survivor or palliative care facilities. Nuclear Survival Centers (NSCs) would be for fixed/hospital-based facilities to optimize survival opportunities and mitigate secondary indirect mortality and morbidity. Nuclear Palliative Care Centers (NPCCs) would be for both fixed and mobile facilities to provide palliative care including pain relief/management, social, psychological, family and burial support services. Health System Support Centers: (HSSCs) would be located in unaffected zones and would endeavor to serve evacuees to recover, restore, rehabilitate and sustain essential public health infrastructure and health systems and to ensure both availability and access to health care in mitigating indirect mortality and morbidity.

Nuclear tragedies are defined by the extremes of population-based medicine where providers must assess and target a population, not individuals, and implement and evaluate interventions that are designed to improve the health of that population efficiently and effectively. Whereas the individual needs are recognized they are not central to the needs of the population’s survival. They are initially managed by health providers who are trained in individually-based care. For most, this will be their first experience in population-driven care and the triage management decisions that will increasingly define how to obtain the best survival outcomes for a population, not the individual casualty directly under their immediate care [23]. In population-based medicine the level of demand outpaces the available supply of resources in a system impaired to the point of near non-operational failure due to overwhelmingly need. Currently, hospitals can usually function for 96 h without external resources. This will be markedly shortened in any nuclear event. Available supplies (e.g., a single surgical pack for multiple victims) will be maximized as will implementing reverse triage decisions which has the advantage of determining which casualties can be safely self-treated.

Triage management will eventually define those in the population who have the best “opportunity for survival” given available resources, and actionable decisions that improve survival outcomes. The extent of the nuclear event, once its parameters are more fully recognized, will more clearly identify the population who have the potential for survival, how they need to be safely managed, with what resources, all of which can be measured. This will minimize direct mortality and morbidity. As this process develops, a multidisciplinary and transdisciplinary governmental body working internationally must exist that represents the sustaining needs for medical care to the defined triage categories, public health protections and infrastructure, security and safety protection enforcements, transportation and rapid decision making. In all wars, over time more mortality occurs from destroyed public health infrastructure and protections (up to 70–90%) than deaths from direct weaponry [24]. This is rarely appreciated and must become a priority as recovering triage categories become increasingly dependent on these protections [13].

## 5. Conclusions

Planning for urban nuclear detonations requires a programmatic approach to the distribution of limited resources for the massive medical and public health needs, which injury category distribution estimation has a significant potential to address. The example of a nuclear strike on Delhi demonstrates this capability, which is useful in principle in extrapolation to large urban centers worldwide. In addition to the widespread loss of life at the local level, there will also be a significant global impact in terms of environmental damage, economic upheaval, and the psychological trauma associated with nuclear war. This approach clearly shows the necessity to bolster India’s medical response and emergency management capabilities, and the need to plan for the distribution of resources to treat millions of nuclear attack survivors in the critical immediate days after an attack. These approaches also show ways to take definitive steps that can be taken to move the nation’s capability much further in the direction of saving lives and alleviating suffering. Our research indicates that millions of people could be potentially saved from fallout radiation exposure by the simple act of evacuating in a timely manner, which would require a robust transportation infrastructure, efficient communication strategies, and most importantly, the interdisciplinary cooperation of medical care, public health, law enforcement, transportation, and national military personnel [22]. Emergency response planners at the local, regional, national, and international level can receive valuable insight into the resource utilization likely to be needed for mobilization in these events. Interestingly, there is a significant potential for political and industry thought leaders to utilize these types of planning outputs to make critical future decisions in their respective arenas. Directly in the emergency response planning, the establishment of emergency preparedness with RTR sites can accomplish a great deal in mobilizing existing and potentially future resources for maximizing emergency response with appropriate mass casualty triage approaches. Therefore, it is in the best interest of India and indeed each potential target nation to rapidly develop and upgrade its inadequate infrastructure which would be beneficial during peacetime, which would also be expected to end up saving millions of lives in the aftermath of a nuclear attack.

## Figures and Tables

**Table 1:** Radii of effect circles for 25 kt and 1 mt weapon.

Effect	Radius (in Meters)	
	25 kt	1 mt
0.6 psi	6185	21,335
1st degree burn	4223	14,531
2nd degree burn	3112	11,880
3rd degree burn	2555	10,332
2 psi	2527	8760
50% Mass Fire	2081	8099
3 psi	1912	6632
4.9 psi	1397	4836
Prompt radiation	1248	2564 *
8.1 psi	1037	3591 *

* Note that for 1 mt weapon, 8.1 psi radius > prompt radiation radius (in 25 kt weapon, 8.1 psi radius < prompt radiation radius).

**Table 2:** Thermal fluence levels for 25 kt and 1 mt nuclear weapon.

Thermal Effect	Thermal Fluence (cal/cm^2^)	
	25 kt	1 mt
1st degree burns	2.5	3.16
2nd degree burns	5	6.21
3rd degree burns	7.71	9.56
50% probability of mass fires	12.01	19.2

**Table 3:** Fallout radiation categories and associated effects.

Fallout Radiation Dose (Gy)	Effects
>10	Fatal—even with intensive management in a normal resource environment
>6–10	Severe radiation injury—hematopoietic and GI syndromes predominate
>2–6	Moderate radiation injury—after a latency period of days to weeks, hematopoietic syndrome is the primary manifestation, followed by the GI syndrome
>0.5–2	Minimal B radiation injury—produces only mild prodromal symptoms
<0.5	Minimal A radiation injury—no immediate symptoms

**Table 4:** Human casualties due to combined injuries (1 × 25 kt nuclear weapon).

Fallout Radiation (Gy)/Effect(s)	0	0.01 to 0.5	>0.5 to 2	>2 to 6	>6 to 10	>10	Total
0.6 psi only	1,219,277	118,470	49,605	58,228	36,303	81,600	1,563,483
0.6 psi + 1st deg burns	652,899	65,626	21,200	21,988	10,334	60,302	832,349
0.6 psi + 2nd deg burns	253,400	29,063	8057	6148	2423	22,861	321,952
0.6 psi + 3rd deg burns	11,272	1071	353	237	93	1177	14,203
2 psi + 3rd deg burns	160,919	16,628	5208	3444	1552	16,601	204,352
2 psi + 50% MF	51,191	6844	1857	1100	463	4939	66,394
3 psi + 50% MF	114,742	18,570	4296	2839	1102	11,775	153,324
4.9 psi + 50% MF	28,420	5407	1642	1205	392	3748	40,814
4.9 psi + Prompt	31,717	8905	2384	2121	741	5951	51,819
8.1 psi + Prompt + 50% MF	18,657	29,396	9946	9035	3821	39,969	110,824
Total	2,542,494	299,980	104,548	106,345	57,224	248,923	3,359,514

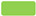
 Minimal; 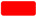 Immediate; 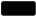 Expectant; 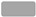 Not accessible for triage.

**Table 5:** Human casualties from fallout radiation only (1 × 25 kt nuclear weapon).

Fallout Radiation Dose (Gy)	Triage Category Allocation for Radiation Injuries Only	Population at 4 h	Population at 24 h
>10	Expectant	4047	47,557
>6–10	Expectant	59,800	129,924
>2–6	Immediate	484,838	564,803
>0.5–2	Minimal B	520,660	601,079
<0.5	Minimal A	1,757,924	2,475,654
Total		2,827,269	3,819,017

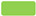
 Minimal; 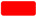 Immediate; 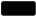 Expectant.

**Table 6:** Human casualties due to combined injuries (6 × 25 kt nuclear weapons).

Fallout Radiation (Gy)/Effect(s)	0	0.01 to 0.5	>0.5 to 2	>2 to 6	>6 to 10	>10	Total
0.6 psi only	515,660	362,583	657,815	1,150,749	608,662	427,857	3,723,326
0.6 psi + 1st deg burns	318,209	160,104	344,176	632,551	147,865	263,831	1,866,736
0.6 psi + 2nd deg burns	147,959	40,854	136,238	245,202	68,743	129,155	768,151
0.6 psi + 3rd deg burns	7408	2338	6430	10,872	3164	6775	36,987
2 psi + 3rd deg burns	104,884	42,358	96,690	150,381	52,266	113,084	559,663
2 psi + 50% MF	27,382	25,493	33,038	50,086	19,422	46,675	202,096
3 psi + 50% MF	7663	152,098	64,176	144,160	30,434	163,022	561,553
4.9 psi + 50% MF	0	24,562	25,769	43,284	7086	46,324	147,025
4.9 psi + Prompt	0	3288	42,141	67,071	13,096	66,371	191,967
8.1 psi + Prompt + 50% MF	0	0	2590	54,862	48,875	298,852	405,179
Total	1,129,165	813,678	1,409,063	2,549,218	999,613	1,561,946	8,462,683

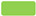
 Minimal; 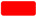 Immediate; 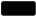 Expectant; 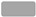 Not accessible for triage.

**Table 7:** Human casualties from fallout radiation only (6 × 25 kt nuclear weapons).

Fallout Radiation Dose (Gy)	Triage Category Allocation for Radiation Injuries Only	Population @ 4 h	Population @ 24 h
>10	Expectant	31,932	546,561
>6–10	Expectant	784,039	1,612,566
>2–6	Immediate	2,768,985	3,039,286
>0.5–2	Minimal B	3,347,265	3,225,508
<0.5	Minimal A	3,492,934	4,759,268
Total		10,425,155	13,183,189

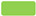
 Minimal; 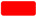 Immediate; 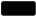 Expectant.

**Table 8:** Human casualties due to combined injuries (1 mt nuclear weapon).

Fallout Radiation (Gy)/Effect(s)	0	0.01 to 0.5	>0.5 to 2	>2 to 6	>6 to 10	>10	Total
0.6 psi only	3,155,533	549,393	131,409	294,732	102,470	3,904	4,237,441
0.6 psi + 1st deg burns	2,286,990	418,080	138,514	83,966	102,018	237,913	3,267,481
0.6 psi + 2nd deg burns	1,318,048	436,686	147,973	82,526	56,590	286,396	2,328,219
0.6 psi + 3rd deg burns	1,061,438	612,034	170,504	61,413	72,198	455,910	2,433,497
2 psi + 3rd deg burns	289,637	281,618	66,262	35,773	18,293	219,834	911,417
2 psi + 50% MF	181,937	825,575	156,196	127,415	48,757	422,301	1,762,181
3 psi + 50% MF	0	708,072	134,413	92,676	40,831	563,713	1,539,705
4.9 psi + 50% MF	0	109,800	293,806	114,560	54,854	341,396	914,416
8.1 psi + 50% MF	0	0	64,324	198,124	70,136	350,380	682,964
8.1 psi + Prompt	0	0	0	0	10,990	635,355	646,345
Total	8,293,583	3,941,258	1,303,401	1,091,185	577,137	3,517,102	18,723,666

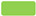
 Minimal; 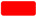 Immediate; 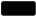 Expectant; 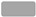 Not accessible for triage.

**Table 9:** Human casualties from fallout radiation only (1 mt nuclear weapon).

Fallout Radiation Dose (rems)	Triage Category Allocation for Radiation Injuries Only	Population @ 4 h	Population @ 24 h
>1000	Expectant	0	0
>600–1000	Expectant	0	65,817
>200–600	Immediate	87,780	1,044,833
>50–200	Minimal B	876,304	611,845
<50	Minimal A	1,235,546	8,770,112
Total		2,199,630	10,492,617

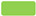
 Minimal; 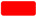 Immediate; 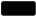 Expectant.

## Data Availability

The data presented in this study are available on request from the corresponding author. The data are not publicly available due to confidentiality concerns.
